# Multiplexed Dilute-and-Shoot Liquid Chromatography–Multiple-Reaction Monitoring Mass Spectrometry Clinical Assay for Metanephrines and Catecholamines in Human Urine

**DOI:** 10.3390/metabo15010030

**Published:** 2025-01-08

**Authors:** Deema O. Qasrawi, Adriano M. C. Pimenta, Evgeniy V. Petrotchenko, Shaun Eintracht, Christoph H. Borchers

**Affiliations:** 1 Segal Cancer Proteomics Centre, Lady Davis Institute for Medical Research, Jewish General Hospital, McGill University, Montreal, QC H3T 1E2, Canada; deema.qasrawi@ladydavis.ca (D.O.Q.); adriano.pimenta@ladydavis.ca (A.M.C.P.); evgeniy.petrotchenko@ladydavis.ca (E.V.P.); 2 Department of Medicine, Sir Mortimer B. Davis Jewish General Hospital, McGill University, Montreal, QC H4A 3J1, Canada; shaun.eintracht.med@ssss.gouv.qc.ca; 3 Division of Clinical and Translational Research, McGill University, Montreal, QC H4A 3J1, Canada; 4 Gerald Bronfman Department of Oncology, Sir Mortimer B. Davis Jewish General Hospital, McGill University, Montreal, QC H3T 1E2, Canada; 5 Segal Cancer Centre, Lady Davis Institute for Medical Research, Sir Mortimer B. Davis Jewish General Hospital, Montreal, QC H3A 2B4, Canada; 6 Department of Pathology, Sir Mortimer B. Davis Jewish General Hospital, Montreal, QC H3A 2B4, Canada

**Keywords:** liquid chromatography–multiple-reaction monitoring mass spectrometry (LC-MRM-MS), metanephrines, catecholamines, adrenal gland, pheochromocytoma, urine, paraganglioma, dilute-and-shoot

## Abstract

**Background:** Quantifying urinary catecholamines and metanephrines is essential for the clinical screening and diagnosis of neuroendocrine tumours. HPLC with electrochemical detection (HPLC-ECD) is commonly used for this type of analysis but requires extensive sample cleanup. Simple and rapid dilute-and-shoot LC–multiple-reaction monitoring (MRM)-MS assays have been developed for quantitating these analytes in urine but have not yet been validated according to the Clinical and Laboratory Standards Institute (CLSI) guidelines. **Methods:** A simple dilute-and-shoot sample preparation without derivatization was used. C18 RP-UPLC-MRM-MS and positive-ion ESI were used, usually with two transitions per analyte being monitored. Certified deuterated internal standards were used for each analyte. **Results:** This assay was validated according to the CLSI C62-A guidelines, including accuracy/trueness, imprecision, sensitivity, specificity, carryover, stability, and linearity. The final MRM-MS method was compared to the established HPLC-ECD clinical chemistry reference method. The run time was reduced from 25 min to 5 min. **Conclusions:** A simple, robust, rapid, and cost-effective LC-MRM-MS assay for measuring urinary catecholamines and metanephrines was developed and validated according to the CLSI guidelines. This validated method requires minimal sample manipulation before analysis and provides sensitivity, specificity, and improved precision. The implementation of this assay in clinical laboratories will facilitate early and accurate diagnosis.

## 1. Introduction

Elevated levels of catecholamines in urine have been linked to a variety of neuroendocrine pathological conditions such as pheochromocytoma (PHEO), paragangliomas (PGLs), and neuroblastoma (NB) [1]. The catecholamines’ norepinephrine (NE), epinephrine (EP), and dopamine (DA) are metabolized to normetanephrine (NMN), metanephrine (MN), and 3-methoxytyramine (3-MT), respectively [2,3,4].

The “gold standard” diagnostic strategy relies on determining the levels of catecholamines in plasma and/or urine, determining the concentrations of metanephrines in plasma, or determining the concentrations of catecholamines and/or their 3-O-methylated metabolites in 24 h urine samples. Measuring catecholamines in urine is challenging due to their low physiological concentrations, the instability of the catechol group, and the need to reduce the pH of the 24 h urine collection [1,5,6]. Several diverse biochemical approaches have been used to quantify catecholamines in biological fluids, including RIA [7], ELISA, voltammetry [8], and thermal-lens spectrometry [9]. Various technical limitations are associated with these methods, however, such as a lack of robustness and sensitivity, false-positive results due to low specificity, the complexity of the setup, high costs, and their time-consuming nature [10,11,12,13].

HPLC with electrochemical detection (ECD) has emerged as a powerful quantitative tool to analyze urinary catecholamines and metanephrines [1,14,15,16]. HPLC-ECD generally requires relatively large sample volumes, extensive sample preparation, and long analytical run times, with the potential for co-eluting interferences [17]. HPLC-ECD also requires frequent calibration to compensate for fluctuations in HPLC pumping rates and the drift of the electrode over time, which can result in increased signal-to-noise ratios [5,18]. SPE is the most common method for extracting metabolites from urine, and this method exhibits both high selectivity and high recovery rates [19]. However, SPE is costly, which is a drawback for its use in routine clinical assays [20].

In contrast, LC-MS methods have a higher sample throughput and a high level of specificity, especially when MRM with labelled internal standards (ISs) is used for data acquisition, reducing the risk of false-positives [21]. Previous studies have used several indirect or in situ derivatization strategies to improve the sensitivity of mass spectrometric detection and the chromatographic separation of catecholamines [22,23]. These assays, however, are still not ideal for routine clinical laboratory and diagnostic purposes because they are time-consuming [23,24,25].

The dilute-and-shoot method is a simple sample clean-up technique where the samples are diluted with an internal standard mixture solution or with a suitable dilution solvent (diluent) for direct analysis [26]. These methods have been recently introduced and have an advantage over other sample clean-up procedures by improving the turnaround time (TAT) and cost-effectiveness since they require only minimal amounts of labour and consumables [19,27,28,29]. In addition, the dilution of urine samples minimizes the salt concentration and reduces matrix effects. Importantly, the decrease in the concentration of the analytes resulting from sample dilution can often be overcome by the high sensitivity of the mass spectrometer [26,27].

Recently, several dilute-and-shoot methods have been described for analyzing catecholamines and metanephrines in the urine. Clark et al. developed an LC-MRM-MS method for the quantitation of vanillylmandelic acid (VMA) and homovanillic acid (HVA) in urine, but this method was not fully validated according to the CLSI guidelines [29]. Yan et al. used LC-MRM-MS for the quantitation of 10 analytes in urine, including EP, DA, and MN, as well as GABA, tryptophan, kynurenine, kynurenic acid, anthranilic acid, glutamic acid, and serotonin, but CLSI validation was not performed [19]. Xie et al. quantitated EP, NE, DA, MN, NMN, 3-MT, serotonin (5-HT), 5-Hydroxyindole-3-acetic acid (5-HIAA), VMA, and HVA in urine using LC-MRM-MS [28], but this method was also not validated according to the CLSI guidelines. The CLSI guidelines aim for the introduction of analytical methods to clinical chemistry practice. They differ from the FDA guidelines on bioanalytical method validation mainly by using definitions and criteria that are commonly accepted in clinical chemistry and comparing new methods with those currently employed in clinical chemistry practice’s reference measurement procedures.

The goal of our study was to validate a simple and rapid LC-MRM-MS method for the highly sensitive and specific quantitation of six clinically relevant catecholamines and metanephrines in urine that would be suitable for clinical use for the detection of elevated levels of analytes in cases of neuroendocrine tumors.

## 2. Materials and Methods

### 2.1. Materials

Certified reference standards (catecholamine mix 1 (epinephrines), catecholamine mix 2 (metanephrines), dopamine, and 3-methoxytyramine) and certified deuterated internal standards (epinephrine-d6, norepinephrine-d6, dopamine-d4, metanephrine-d3, normetanephrine-d3, and 3-methoxytyramine-d4) were purchased from Cerilliant Corporation (Round Rock, TX, USA). LC-MS-grade ACN, water, and formic acid were purchased from Sigma-Aldrich (St. Louis, MO, USA). Catecholamine-free urine (Mass Spect Gold Human Urine, Catecholamine Free) was purchased from Golden West Biologicals Inc (Temecula, CA, USA). Lyphocheck Quantitative Controls Level I and Level II and “Catecholamines and Metanephrines by HPLC Urine Calibrator” were purchased from Bio-Rad Laboratories, Inc. (Hercules, CA, USA).

#### 2.1.1. Urine Sample Collection

The 96 patients’ 24 h urine samples included in this study were submitted to the Jewish General Hospital clinical chemistry laboratory for testing for metanephrines and catecholamines. The samples were collected into 24 h urine collection containers with 50 mL of 3.4 M HCl added. The fractionated catecholamines and metanephrines were measured by HPLC-ECD, using the method used as the routine clinical HPLC-ECD method at the Jewish General Hospital. Approval was obtained from the Institutional Review Board (IRB) Ethics Committee of the Jewish General Hospital, and this approval was in place as part of the quality assurance program. This research was performed in accordance with the Helsinki Declaration.

#### 2.1.2. External Quality Assurance

External quality assurance samples from Randox Laboratories Limited (Crumlin, County Antrim, UK) were analyzed in the Jewish General Hospital clinical chemistry lab as part of the Randox International Quality Assessment Scheme (RIQAS) to assess accuracy. The leftover material was given to the Segal Cancer Proteomics Centre for analysis.

### 2.2. Methods

#### 2.2.1. Preparation of Calibration Standards and QC Samples

Stock solutions of the non-labelled standards and the deuterated internal standards (ISs) were prepared at various concentrations with 0.1% formic acid in water, and aliquots were stored at −80 °C. Working solutions for an 8-point standard curve were generated by appropriate dilutions of the stock solutions in Mass Spect Gold Urine. Calibration curves were generated using 8 points at concentrations of 79.2, 158.5, 317, 634, 1267.8, 2535.5, 5071, and 10,142 nM for the MN; 85.3, 170.6, 341.2, 682.3, 1364.6, 2729.3, 5458.5, and 10,917 nM for the NMN; 93.5, 187, 374, 747.6, 1495.2, 2990.4, 5980.9, and 11,961.72 nM for the 3-MT; 85.3, 170.6, 341.2, 682.3, 1364.6, 2729.3, 5458.5, and 10,917 nM for the EP; 92.3, 184.7, 369.4, 738.8, 1477.5, 2955.1, 5910.2, and 11,820.3 nM for the NE; and 102, 204, 408, 816, 1631.8, 3263.7, 6527.4, and 13,054.8 nM for the DA spiked into catecholamine-free urine. Linearity was determined by plotting the ratio of the analyte peak area to the IS area vs. the concentration of the standards, using weighted linear regression (1/x).

Three quality control samples (low, medium, and high) were prepared at concentrations of 93.75, 750, and 1500 µg/L, which were equal to 475.4, 3803.3, and 7606.5 nM for the MN; 511.7, 4093.9, and 8187.8 nM for the NMN; 560.7, 4485.7, and 8971.3 nM for the 3-MT; 511.7, 4093.9, and 8187.8 nM for the EP; 554.1, 4432.6, and 8865.3 nM for the NE; and 612, 4895.6, and 9791.1 nM for the DA spiked into catecholamine-free urine.

In addition, for the comparison to the HPLC-ECD reference method, a calibrator and two quality control samples for urinary catecholamines and metanephrines were prepared using the Lyphocheck Quantitative Urine Control. Briefly, the lyophilized calibrator and Quality Control I and II samples prepared from human urine were spiked with standards and reconstituted with 10 mL of 0.05M HCl. Quality Control I and Quality Control II represented normal and abnormal samples within the clinical range. The calibrator, QC I, and QC II mean concentrations were listed as follows: EP, 475, 71.4, and 437; NE, 1100, 234, and 437; DA, 1874, 436, and 3454; MN, 1947, 504, and 2602; NMN, 3809, 1793, and 8105; and 3-MT, 2063, 339, and 3346 nM, respectively. Aliquots of the calibrator and quality control samples were stored at −80 °C. Both the calibrator and quality control samples were prepared and treated in the same manner as the patient’s urine samples.

#### 2.2.2. Sample Preparation

A 180 µL aliquot of the internal standard mixture (0.5 ng/mL each, prepared in 0.1% formic acid in water) was added to a 20 µL aliquot of each calibrator, quality control, or urine sample in a 2 mL microcentrifuge tube and vortexed. The tubes were centrifuged at 21,100× *g* for 1 min at 22 °C. A 60 µL aliquot of the supernatant from each tube was transferred into a conical glass insert assay vial and placed into the LC-MS autosampler for analysis.

#### 2.2.3. LC-MRM-MS Analysis

LC-MRM-MS analysis was performed using an ultra-high performance liquid chromatography (UHPLC) Nexera XR UHPLC system (Shimadzu, Kyoto, Japan) coupled to a Sciex QTrap 6500+ mass spectrometer using the IonDrive Turbo V ESI source (Framingham, MA, USA). Chromatographic separation was performed using a Zorbax Eclipse Plus C18 column (2.1 × 150 mm, 1.8 μm 95 Å particles, Agilent Technologies, Mississauga, ON, Canada) with an in-line filter and a 5 min gradient separation program (mobile phase A: 0.1% formic acid in HPLC-grade water; mobile phase B: 0.1% formic acid in ACN). The LC separations were performed at a flow rate of 0.400 mL/min, with a gradient starting at 0% B for 0.25 min and then ramping up to 25% B between 0.25 and 2.5 min. The % B was then ramped up to 95% between 2.5 and 3.0 min. The % B was ramped back to the initial conditions (0%) between 3.0 and 3.1 min and held at 0% B until 5.0 min. The sample injection volume was 1 µL, the column oven was set to 45 °C, and the autosampler temperature was set to 15 °C.

Sample ionization was performed using positive-ion ESI. The source parameters were optimized as follows: the capillary voltage was 5.5 kV; the source temperature was 500 °C; ion source gas 1 was set to 60; ion source gas 2 was set to 50; the collision gas was set to high; and the curtain gas was set to 30. Data acquisition was performed using Sciex’s Analyst 1.6 software, using multiple-reaction monitoring (MRM) to monitor two transitions (a qualifier and a quantifier) for each analyte and its internal standard, except for the NE and NE-d6, where we were not able to find a second good transition to monitor. The quantifier–qualifier ratio was monitored to confirm analyte identification and was considered acceptable if it was within 20% of the expected value. The MRM transitions and optimized MS parameters are listed in Table 1. The ratio of the chromatographic peak area of each analyte’s quantifier transition to that of the quantifier transition for its deuterated internal standard was used for quantitation.

#### 2.2.4. HPLC-ECD Analysis

The HPLC-ECD analyses were performed at the clinical laboratory at the Jewish General Hospital, using their standard analytical method [30].

#### 2.2.5. Method Validation

The developed method was evaluated using the CLSI guidelines (CLSI C62-A) for linearity, sensitivity, carryover, and intra-day and inter-day imprecision [31]. The trueness/accuracy of the new LC-MS method was assessed using three different approaches [32]. (i) Method comparison: levels of catecholamines and metanephrines were measured from 24 h urine samples from 96 patients and compared to the results obtained by the clinical laboratory using their routine HPLC-ECD method. (ii) Materials with assigned values: samples from the RIQAS Human Urine External Quality Assurance program were submitted to the Jewish General Hospital’s clinical chemistry laboratory (Supplementary Table S1) for metanephrine and catecholamine analysis. (iii) Spiking analysis: the average percent recovery from the spiked catecholamine-free urine was evaluated for four levels for each analyte (n = 4) using certified reference standards from Cerilliant^®^ (Round Rock, TX, USA).

#### 2.2.6. Data Analysis

Chromatographic data were collected and integrated using Analyst software version 1.3.2 from Sciex. Calibration curves were constructed based on the plotting of the analyte-to-IS peak area ratio versus the concentration of the standard. The concentrations in unknown samples were calculated from the best-fit linear equation (y = ax + b), where x is the analyte concentration and y is the peak area ratio of the analyte to IS. All statistical analyses were performed using Microsoft Excel 2016.

## 3. Results

The goal of this study was to develop a simple, robust, and sensitive LC-MS method for implementation in clinical chemistry practice. We utilized a combination of minimal sample preparation and robust, standard, C18-based, reversed-phase chromatography combined with MRM-MS detection and the use of certified deuterated internal standards.

**Figure 1:**
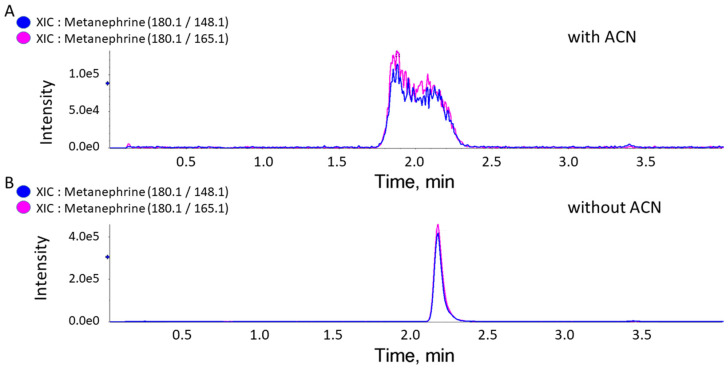
Extracted ion chromatograms (XICs) for MN with and without ACN. XICs for metanephrine’s two transitions (quantifier and qualifier) showing a poor peak shape obtained when ACN was used in the dilution step. (**A**) An XIC with ACN in the dilution step. (**B**) An XIC without ACN, using only 0.1% FA in the dilution step.

Using ACN (final extract *v*/*v* concentrations of 50 to 80%) in our multiplexed dilute-and-shoot assay for NE, EP, DA, NMN, MN, and 3-MT [2,3,4] generated an improper peak shape for MN (Figure 1). Our improved dilute-and-shoot LC-MRM-MS method, without ACN in the sample, allowed the analysis of all six clinically relevant analytes in a single analysis and was validated according to the CLSI guidelines. The resulting dilute-and-shoot LC-MRM-MS method is simple, rapid, cost-effective, and not labor-intensive and is suitable for clinical chemistry use.

**Figure 2:**
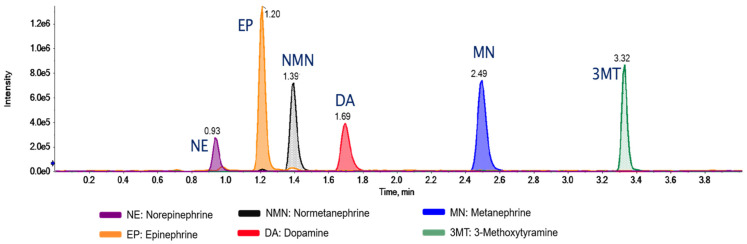
Extracted ion chromatograms (XICs). XICs were obtained for the multiplexed detection of norepinephrine (NE), epinephrine (EP), normetanephrine (NMN), dopamine (DA), metanephrine (MN), and 3-methoxytyramine (3MT), using a reversed-phase C18 column, in a spiked catecholamine-free urine sample (250 µg/L each). The response of the analytes is expressed as intensity (y-axis) vs. time (x-axis).

To summarize this procedure, urine samples were diluted with IS solutions and centrifuged and the supernatants were analyzed by LC-MRM-MS. No derivatization or sample cleanup was required, resulting in a simple, rapid, and robust dilute-and-shoot method. The catecholamine and metanephrine analytes (and their internal standards) were measured using a QTRAP mass spectrometer in the MRM mode using positive-ion ESI. Precursor ions for MN and NMN showed spontaneous water loss (−18 Da) followed by methoxy group loss (−32 Da), leading to intense product–ion transitions, in agreement with previous studies [17,33,34,35] (Supplementary Figure S1). The reversed-phase C18 LC conditions resulted in a baseline separation for the six analytes within a total run time of 5.0 min (Figure 2 and Supplementary Figure S2).

The performance of the method was evaluated and validated according to the CLSI guidelines and included the determination of linearity, sensitivity, imprecision, trueness, and carryover. The results are summarized in Table 2. The linearity of the standard curve was assessed by analyzing calibrators prepared in catecholamine-free urine at eight concentration levels using formula y = ax + b, where y is the ratio of the analyte peak area to the IS area and x is the analyte concentration. Three replicates of each concentration level were analyzed. The acceptance criterion for linearity was a coefficient of determination (r^2^) ≥ 0.997 (Supplementary Figure S3). Carryover was assessed by injecting a blank sample following a high-concentration sample, and a carryover < 1.7% of the LLMI was observed (Table 2).

Intra-day imprecision was determined by analyzing each QC sample (i.e., each concentration level) in ten replicates (n = 10) within a single batch. Inter-day imprecision was assessed by measuring each level of QC sample in four replicates over five consecutive days (n = 20). The coefficients of variation (CVs) for the intra-day and inter-day imprecision were 2.1–7.7% and 2.6–13.2%, respectively (Table 3). The stability of the analytes in acidified urine samples has been thoroughly investigated and referenced elsewhere [19,28]. Freezer stability was indirectly assessed by inter-day imprecision measurements, and autosampler stability (15 °C) was assessed for 24 h and found to be satisfactory.

A Bland–Altman and Passing and Bablok regression were used to characterize the mean bias between the HPLC-ECD and LC-MRM-MS measurements and to determine the limits of agreement (a mean bias ±1.96 times the standard deviation; SD) and the underlying variabilities [36,37]. The correlation between the HPLC-ECD and the LC-MS method is shown in Supplementary Figure S4. While some bias was present, an acceptable agreement was observed between the HPLC-ECD and the LC-MRM-MS methods. 3-methoxytyramine (3-MT) was not included because the analytical reference method did not measure this compound. Moreover, the external QC samples showed that the new LC-MS method performed well, with a standard deviation index (SDI) of less than 2 (Supplementary Table S1). The average percent recovery of the reference materials, the third approach for assessing the trueness/accuracy of our assay, was also within the acceptability criteria of ±15% (Table 4) in accordance with the CLSI guidelines.

Clinical Application: During the method-validation portion of this study, we had the opportunity to analyze a urine sample from a 69-year-old male patient with an adrenal mass who was taking antihypertensive medication (Irbesartan) and mesalamine (Mesalazine) for inflammatory bowel disease. The patient submitted a 24 h urine sample for the determination of catecholamine and metanephrine concentrations. Mesalamine is a known interference for normetanephrine by HPLC-ECD, and the urinary normetanephrine level was found to be >10,000 nmol/L by HPLC-ECD and 352 nmol/L by LC-MRM-MS. The adrenal mass was further investigated for other secretory products and by further imaging and was diagnosed and determined to be non-secretory, in agreement with the values determined by the LC-MRM-MS method. This confirmed the improved specificity obtained and the clinical value of using our LC-MRM-MS method compared to HPLC-ECD.

## 4. Discussion

In this study, we developed a simple dilute-and-shoot LC-MS method for use in clinical chemistry laboratories to simultaneously quantify catecholamines and their respective O-methylated metabolites in human urine samples. All compounds were separated and detected using RP-HPLC combined with MRM mass spectrometry in the positive-ion ESI mode. We used two MRM transitions (a quantifier and a qualifier ion) for each analyte and its internal standard, except for norepinephrine (NE and NE-d6), where only one transition was monitored because we were not able to find a suitable second transition. The use of a deuterated internal standard for each analyte compensated for the effects of ion suppression. Additionally, the ion ratios were monitored to ensure the assay’s specificity, validate the accurate identification and quantitation of the analytes, and detect potential interferences. Chromatographic baseline separation for all six compounds was achieved in a 5 min total run time. Although EP/NMN and 3-MT/MN are isobaric analytes and are challenging to separate [38], they were baseline separated under the conditions used. Reducing the run time from 25 min to 5 min was an advantage when moving the assay from HPLC-ECD to an LC-MRM-MS platform.

While dilution is an essential step to reduce matrix effects, it can also hamper analyte detection. The main drawback of dilute-and-shoot methods is their limited sensitivity due to the absence of a pre-concentration step. Fortunately, this problem can be overcome by the new generation of highly sensitive instruments. Urine is a complex matrix containing many compounds, including phospholipids, proteins, salts, urea, and creatinine. The clogging of the chromatographic system is also a risk due to compounds usually present in urine, but this problem can be resolved by centrifugation and in-line filtration.

Using a deuterated internal standard for each compound is an essential feature of our method and it compensates for ion suppression or pipetting imprecision. The robustness of the developed LC-MRM-MS method makes it easily transferable to a clinical laboratory for routine use. Measuring the concentrations of catecholamine and the catecholamine metabolites NE, EP, DA, NMN, MN, and 3-MT combined in a single assay provides additional diagnostic value for identifying tumors that predominantly produce dopamine [39,40]. The Bland–Altman and Passing and Bablok regression showed bias in the results between the two methods. Given that the MS-based detection was superior to ECD (the determined LOQ values (0.3, 0.5, 2.6, 8.5, 2.3, and 4.6 nM for MN, NE, DA, EP, 3-MT, and NMN, respectively) were much lower than those for the HPLC-ECD), correlations between LC-MS and HPLC-ECD should not be directly attributed to the methods’ performance. Additionally, ECD lacks selectivity and specificity. Nevertheless, the differences in values between the two methods are not dramatic, which can insure the meaningful comparison of data between the two methods, for example, in cases of long-term post-surgery monitoring. This assay can also be used for laboratory diagnosis, monitoring, and detecting neuroendocrine tumour recurrence. The main subject of this the study was to compare the new method to the existing reference procedure as a part of the validation of the assay, which is the main difference between the CLSI guidelines and the FDA guidelines [31]. This comparison provided support for the replacement of the method that is currently used in the clinical chemistry laboratory by the LC-MS-based assay.

## 5. Conclusions

A simple dilute-and-shoot LC-MS method for clinical use was developed to simultaneously quantify catecholamines and their respective O-methylated metabolites in human urine samples. The method provided high selectivity, high accuracy and precision, and high throughput, giving it advantages over commonly used methods. In this dilute-and-shoot approach, we minimized the sample volume required, simplified the sample processing, and achieved high-throughput sample preparation for quantifying catecholamines and metanephrines in human urine samples. Replacing the costly and time-consuming SPE step in SPE-based sample clean-up methods with a very simple dilute-and-shoot approach allowed for a considerably higher sample throughput with a ~five-fold reduction in the analysis time. This assay was validated according to the CLSI guidelines, and our analyses showed that it passed the CLSI acceptability criteria. This new assay can be readily implemented in clinical settings and should improve TAT.

## Figures and Tables

**Table 1:** Multiple-reaction monitoring parameters for the six analytes and their internal standards in this study ^a^.

Analyte	Precursor Ion	Quantifier Transition				Qualifier Transition				Ion Ratio
		Product Ion	CE	DP	CXP	Product Ion	CE	DP	CXP	
Metanephrine	180.1	148.1	23	50	13	165.1	23	50	13	1.24
Normetanephrine	166.1	134.1	23	50	13	149.1	23	50	13	0.38
3-Methoxytyramine	151.2	91.2	25	108	13	119.1	19	108	13	1.53
Epinephrine	184.2	166.2	15	20	3	107.2	20	20	13	0.11
Norepinephrine	152.2	135.2	15	50	13	__	__	__	__	NA *
Dopamine	154.1	91.1	31	20	3	137.1	30	20	3	0.18
Metanephrine-*d3*	183.1	151.1	23	50	13	168.1	23	50	13	0.99
Normetanephrine-*d3*	169.1	137.1	23	50	13	109.1	23	50	13	0.56
3-Methoxytyramine-*d4*	155.1	95.1	25	108	13	123.1	19	108	13	1.9
Epinephrine-*d6*	190.2	172.1	15	20	3	113.2	30	20	13	0.04
Norepinephrine-*d6*	158.2	139.2	15	50	13	__	__	__	__	NA *
Dopamine-*d4*	158.1	95.1	31	20	3	141.1	30	20	3	2.07

^a^: CE: collision energy; DP: declustering potential; CXP: cell exit potential; * NA: only one transition was monitored for norepinephrine.

**Table 2:** Summary of linearity, sensitivity, and carryover.

Compound	Measuring Interval (nmol/L)	Slope (a)	Intercept (b)	Coefficient of Determination (r^2^)	Carryover (%)
Metanephrine	79.2–10,100	0.0008	+0.0122	1.0000	0.0
Normetanephrine	85.3–10,917	0.0009	−0.0323	0.9998	0.8
3-Methoxytyramine	93.5–11,962	0.0014	−0.0004	1.0000	0.0
Epinephrine	85.3–10,917	0.0007	+0.0274	1.0000	0.0
Norepinephrine	92.3–11,820	0.0011	−0.0481	0.9997	1.7
Dopamine	101.9–13,055	0.0011	−0.0233	0.9999	0.0

**Table 3:** Coefficients of variation (CVs) for intra-day and inter-day imprecision. CVs for intra-day and inter-day imprecision at four different concentrations (LLMI, QCL, QCM, and QCH) in spiked catecholamine-free urine samples.

Compound	CV (%) Intra-Day (n = 10)				CV (%) Inter-Day (n = 20)			
	LLMI	QCL	QCM	QCH	LLMI	QCL	QCM	QCH
Metanephrine	5.38	2.74	3.48	4.84	11.79	3.80	3.58	2.63
Normetanephrine	7.66	3.36	3.50	3.70	12.06	3.22	4.96	3.04
3-Methoxytyramine	2.15	2.48	2.44	3.07	4.85	3.13	4.22	4.23
Epinephrine	3.50	3.01	2.39	3.45	13.23	4.09	3.62	3.33
Norepinephrine	5.94	7.58	7.19	3.09	11.25	6.61	3.87	3.83
Dopamine	2.06	3.34	5.30	5.17	7.00	5.77	3.91	2.83

Abbreviations: LLMI: lower limit of measurement interval; QCL: quality control low; QCM: quality control medium; QCH: quality control high.

**Table 4:** Reference material (% recovery). Average % recovery of reference material in spiked catecholamine-free urine samples for four levels for each analyte (n = 4) using certified reference standards from Cerilliant was within acceptability criteria of ±15%.

MN					NMN					3-MT				
	Level 1	Level 2	Level 3	Level 4		Level 1	Level 2	Level 3	Level 4		Level 1	Level 2	Level 3	Level 4
Target conc. [nM]	79.2	475	3800	7610	Target conc. [nM]	85.3	512	4090	8190	Target conc. [nM]	92.3	554	4430	8870
Avg. measured conc. [nM] (n = 4)	78.0	489.5	3787.5	7525.0	Avg. measured conc. [nM] (n = 4)	86.4	505.0	3985.0	8177.5	Avg. measured conc. [nM] (n = 4)	87.2	556.3	4412.5	8777.5
Recovery %	97.6	103.9	98.9	99.3	Recovery %	100.7	97.9	97.1	99.5	Recovery %	95.1	101.6	99.2	100.3
Recovery % (avg.)	100.02				Recovery % (avg.)	99.30				Recovery % (avg.)	98.40			
EP					NE					DA				
	Level 1	Level 2	Level 3	Level 4		Level 1	Level 2	Level 3	Level 4		Level 1	Level 2	Level 3	Level 4
Target conc. [nM]	85.3	512	4090	8190	Target conc. [nM]	102	612	4900	9790	Target conc. [nM]	93.5	561	4490	8970
Avg. measured conc. [nM] (n = 4)	83.6	524.0	4302.5	8235.0	Avg. measured conc. [nM] (n = 4)	87.2	556.3	4412.5	8777.5	Avg. measured conc. [nM] (n = 4)	92.2	616.0	5002.5	10,177.5
Recovery %	102.3	102.9	106.4	100.0	Recovery %	86.0	91.9	89.7	90.9	Recovery %	96.4	108.4	111.7	113.4
Recovery % (avg.)	101.50				Recovery % (avg.)	89.00				Recovery % (avg.)	108.30			

## Data Availability

Dataset available on request from the authors.

## Supplementary Materials

The following supporting information can be downloaded at: https://www.mdpi.com/article/10.3390/metabo15010030/s1, Supplementary Figure S1. MRM transition of NMN; Supplementary Figure S2. Evaluation of peak resolution; Supplementary Figure S3. Calibration standard curve; Supplementary Figure S4. Bland-Altman plot (A) and Passing-Bablok regression (B) for the results from the new LC/MRM-MS method versus the HPLC-ECD; Supplementary Table S1. Accuracy assessment using EQA samples; Supplementary Table S2. Concentration values (nM) measured by HPLC-ECD and by LC-MRM-MS methods.

## Abbreviations

ACN—acetonitrile; CLSI—Clinical and Laboratory Standards Institute; CV—coefficient of variation; DA—dopamine; ECD—electrochemical detection; EP—epinephrine; LC-MRM-MS—liquid chromatography–multiple-reaction monitoring mass spectrometry; MN—metanephrine; MRM-MS—multiple-reaction monitoring mass spectrometry; 3-MT—3-methoxytyramine; NB—neuroblastoma; NE—norepinephrine; NMN—normetanephrine; PHEO—pheochromocytoma; PGLs—paragangliomas; RIQAS—Randox International Quality Assessment Scheme; TAT—turnaround time; XIC—extracted ion chromatogram.

## References

[1] Grouzmann E., Lamine F. (2013). Determination of catecholamines in plasma and urine. Best Pract. Res. Clin. Endocrinol. Metab..

[2] de Jong W.H., de Vries E.G., Kema I.P. (2011). Current status and future developments of LC-MS/MS in clinical chemistry for quantification of biogenic amines. Clin. Biochem..

[3] Weinkove C. (1991). ACP Broadsheet No 127: April 1991. Measurement of catecholamines and their metabolites in urine. J. Clin. Pathol..

[4] Lenders J.W., Duh Q.Y., Eisenhofer G., Gimenez-Roqueplo A.P., Grebe S.K., Murad M.H., Naruse M., Pacak K., Young W.F., Endocrine S. (2014). Pheochromocytoma and paraganglioma: An endocrine society clinical practice guideline. J. Clin. Endocrinol. Metab..

[5] Peaston R.T., Weinkove C. (2004). Measurement of catecholamines and their metabolites. Ann. Clin. Biochem..

[6] Davies S.L., Davison A.S. (2019). Liquid chromatography tandem mass spectrometry for plasma metadrenalines. Clin. Chim. Acta.

[7] Cooper R.L., Walker R.F. (1983). Microradioenzymic assays for the measurement of catecholamines and serotonin. Methods Enzymol..

[8] Jones S.R., Mickelson G.E., Collins L.B., Kawagoe K.T., Wightman R.M. (1994). Interference by pH and Ca^2+^ ions during measurements of catecholamine release in slices of rat amygdala with fast-scan cyclic voltammetry. J. Neurosci. Methods.

[9] Sanchis-Mallols J.M., Villanueva-Camañas R.M., Ramis-Ramos G. (1992). Determination of unconjugated catecholamine in urine as dopamine by thermal lens spectrometry. Analyst.

[10] Raum W.J. (1984). Methods of plasma catecholamine measurement including radioimmunoassay. Am. J. Physiol..

[11] Qasrawi D.O., Boyd J.M., Sadrzadeh S.M.H. (2021). Measuring steroids from dried blood spots using tandem mass spectrometry to diagnose congenital adrenal hyperplasia. Clin. Chim. Acta.

[12] Georges J. (1999). Advantages and limitations of thermal lens spectrometry over conventional spectrophotometry for absorbance measurements. Talanta.

[13] Smith A.R., Garris P.A., Casto J.M. (2015). Real-time monitoring of electrically evoked catecholamine signals in the songbird striatum using in vivo fast-scan cyclic voltammetry. J. Chem. Neuroanat..

[14] Bertani-Dziedzic L.M., Krstulovic A.M., Dziedzic S.W., Gitlow S.E., Cerqueira S. (1981). Analysis of urinary metanephrines by reversed-phase high-performance liquid chromatography and electrochemical detection. Clin. Chim. Acta.

[15] Lenders J.W.M., Pacak K., Walther M.M., Linehan W.M., Mannelli M., Friberg P., Keiser H.R., Goldstein D.S., Eisenhofer G. (2002). Biochemical Diagnosis of Pheochromocytoma: Which Test Is Best?. J. Am. Med. Assoc..

[16] Davidson F.D., Davidson F.D. (2004). Paracetamol-associated interference in an HPLC-ECD assay for urinary free metadrenalines and catecholamines. Ann. Clin. Biochem..

[17] Peaston R.T., Graham K.S., Chambers E., van der Molen J.C., Ball S. (2010). Performance of plasma free metanephrines measured by liquid chromatography-tandem mass spectrometry in the diagnosis of pheochromocytoma. Clin. Chim. Acta.

[18] Shushan B., Roberts W.L., Frank E.L., Urry F.M., Kushnir M.M. (2002). Analysis of Catecholamines in Urine by Positive-Ion Electrospray Tandem Mass Spectrometry. Clin. Chem..

[19] Yan J., Kuzhiumparambil U., Bandodkar S., Solowij N., Fu S. (2017). Development and validation of a simple, rapid and sensitive LC-MS/MS method for the measurement of urinary neurotransmitters and their metabolites. Anal. Bioanal. Chem..

[20] Strathmann F.G., Hoofnagle A.N. (2011). Current and future applications of mass spectrometry to the clinical laboratory. Am. J. Clin. Pathol..

[21] Kline G.A., Boyd J., Leung A.A., Tang A., Sadrzadeh H.M. (2020). Very high rate of false positive biochemical results when screening for pheochromocytoma in a large, undifferentiated population with variable indications for testing. Clin. Biochem..

[22] Zheng J., Mandal R., Wishart D.S. (2018). A sensitive, high-throughput LC-MS/MS method for measuring catecholamines in low volume serum. Anal. Chim. Acta.

[23] van Faassen M., Bischoff R., Eijkelenkamp K., de Jong W.H.A., van der Ley C.P., Kema I.P. (2020). In Matrix Derivatization Combined with LC-MS/MS Results in Ultrasensitive Quantification of Plasma Free Metanephrines and Catecholamines. Anal. Chem..

[24] van de Merbel N.C., Hendriks G., Imbos R., Tuunainen J., Rouru J., Nikkanen H. (2011). Quantitative determination of free and total dopamine in human plasma by LC-MS/MS: The importance of sample preparation. Bioanalysis.

[25] Zhang G., Zhang Y., Ji C., McDonald T., Walton J., Groeber E.A., Steenwyk R.C., Lin Z. (2012). Ultra sensitive measurement of endogenous epinephrine and norepinephrine in human plasma by semi-automated SPE-LC–MS/MS. J. Chromatogr. B.

[26] Greer B., Chevallier O., Quinn B., Botana L.M., Elliott C.T. (2021). Redefining dilute and shoot: The evolution of the technique and its application in the analysis of foods and biological matrices by liquid chromatography mass spectrometry. TrAC Trends Anal. Chem..

[27] Deventer K., Pozo O.J., Verstraete A.G., Van Eenoo P. (2014). Dilute-and-shoot-liquid chromatography-mass spectrometry for urine analysis in doping control and analytical toxicology. TrAC Trends Anal. Chem..

[28] Xie Z., Lorkiewicz P., Riggs D.W., Bhatnagar A., Srivastava S. (2018). Comprehensive, robust, and sensitive UPLC-MS/MS analysis of free biogenic monoamines and their metabolites in urine. J. Chromatogr. B Anal. Technol. Biomed. Life Sci..

[29] Clark Z.D., Cutler J.M., Pavlov I.Y., Strathmann F.G., Frank E.L. (2017). Simple dilute-and-shoot method for urinary vanillylmandelic acid and homovanillic acid by liquid chromatography tandem mass spectrometry. Clin. Chim. Acta.

[30] Chan E.C., Wee P.Y., Ho P.C. (2000). Evaluation of degradation of urinary catecholamines and metanephrines and deconjugation of their sulfoconjugates using stability-indicating reversed-phase ion-pair HPLC with electrochemical detection. J. Pharm. Biomed. Anal..

[31] Lynch K.L. (2016). CLSI C62-A: A New Standard for Clinical Mass Spectrometry. Clin. Chem..

[32] Wayne P. (2014). CLSI. Liquid Chromatography-Mass Spectrometry Methods.

[33] Taylor R.L., Singh R.J. (2002). Validation of Liquid Chromatography–Tandem Mass Spectrometry Method for Analysis of Urinary Conjugated Metanephrine and Normetanephrine for Screening of Pheochromocytoma. Clin. Chem..

[34] Lagerstedt S.A., O’Kane D.J., Singh R.J. (2004). Measurement of plasma free metanephrine and normetanephrine by liquid chromatography-tandem mass spectrometry for diagnosis of pheochromocytoma. Clin. Chem..

[35] Eisenhofer G., Peitzsch M., McWhinney B.C. (2016). Impact of LC-MS/MS on the laboratory diagnosis of catecholamine-producing tumors. TrAC Trends Anal. Chem..

[36] Clinical and Laboratory Standards Institute (2013). Approved Guideline—Second Edition EP9-A3—Measurement Procedure Comparison and Bias Estimation Using Patient Samples.

[37] Bland J.M., Altman D.G. (1986). Statistical methods for assessing agreement between two methods of clinical measurement. Lancet.

[38] Chan E.C., Ho P.C. (2000). High-performance liquid chromatography/atmospheric pressure chemical ionization mass spectrometric method for the analysis of catecholamines and metanephrines in human urine. Rapid Commun. Mass Spectrom..

[39] Eisenhofer G., Goldstein D.S., Sullivan P., Csako G., Brouwers F.M., Lai E.W., Adams K.T., Pacak K. (2005). Biochemical and clinical manifestations of dopamine-producing paragangliomas: Utility of plasma methoxytyramine. J. Clin. Endocrinol. Metab..

[40] Peitzsch M., Prejbisz A., Kroiss M., Beuschlein F., Arlt W., Januszewicz A., Siegert G., Eisenhofer G. (2013). Analysis of plasma 3-methoxytyramine, normetanephrine and metanephrine by ultraperformance liquid chromatography-tandem mass spectrometry: Utility for diagnosis of dopamine-producing metastatic phaeochromocytoma. Ann. Clin. Biochem..

