# Supplementary material for: Multiplexed Dilute-and-Shoot Liquid Chromatography–Multiple-Reaction Monitoring Mass Spectrometry Clinical Assay for Metanephrines and Catecholamines in Human Urine
Source: Metabolites. 2025 Jan 8;15(1):30. doi: 10.3390/metabo15010030 (PMC11767516; doi:10.3390/metabo15010030)
Supplement: Supplementary file 1 [file metabolites-15-00030-s001.zip › metabolites-3298616-supplementary.pdf]

Multiplexed Dilute-and-Shoot Liquid Chromatography–Multiple-Reaction Monitoring Mass Spectrometry Clinical Assay for Metanephrines and Catecholamines in Human Urine

Deema O. Qasrawi [1], Adriano M. C. Pimenta [1], Evgeniy V. Petrotchenko [1], Shaun Eintracht [2] and Christoph H. Borchers [1,3,4,5,6,*]

[1]Segal Cancer Proteomics Centre, Lady Davis Institute for Medical Research, Jewish General Hospital, McGill University, Montreal, QC H3T 1E2, Canada; deema.qasrawi@ladydavis.ca (D.O.Q.); adriano.pimenta@ladydavis.ca (A.M.C.P.); evgeniy.petrotchenko@ladydavis.ca (E.V.P.)

[2]Department of Medicine, Sir Mortimer B. Davis Jewish General Hospital, McGill University,
Montreal, QC H4A 3J1, Canada; shaun.eintracht.med@ssss.gouv.qc.ca

[3]Division of Clinical and Translational Research,, McGill University, Montreal, QC H4A 3J1, Canada

[4]Gerald Bronfman Department of Oncology, Sir Mortimer B. Davis Jewish General Hospital,
McGill University, Montreal, QC H3T 1E2, Canada

[5]Segal Cancer Centre, Lady Davis Institute for Medical Research, Sir Mortimer B. Davis Jewish General
Hospital, Montreal, QC H3A 2B4, Canada

[6]Department of Pathology, Sir Mortimer B. Davis Jewish General Hospital, Montreal, QC H3A 2B4, Canada

*Correspondence: christoph.borchers@mcgill.ca

***Corresponding author:**
Christoph H. Borchers
Segal Cancer Proteomics Centre
Lady Davis Institute for Medical Research, Jewish General Hospital
3755 Côte Ste. Catherine Road, Room E615
McGill University, Montreal, QC H3T 1E2, Canada
E-mail: christoph.borchers@mcgill.ca
Telephone: 1-514-340-8222, ext. 7886

Supplementary Figures

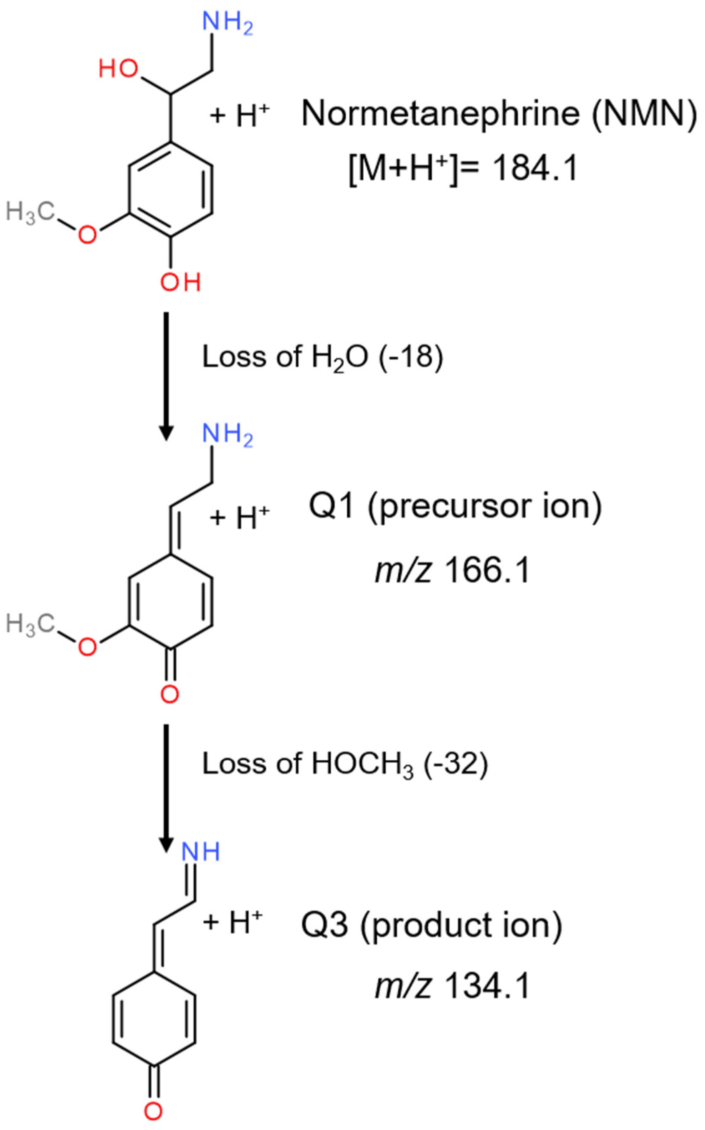

Supplementary Figure S1. MRM transition of NMN. In the positive ESI mode, NMN goes through a spontaneous water loss from the protonated molecular ion, giving fragment *m/z* 166.1. Further loss of a methoxy group in the Q3 gives the major product ion *m/z* 134.1 [1].

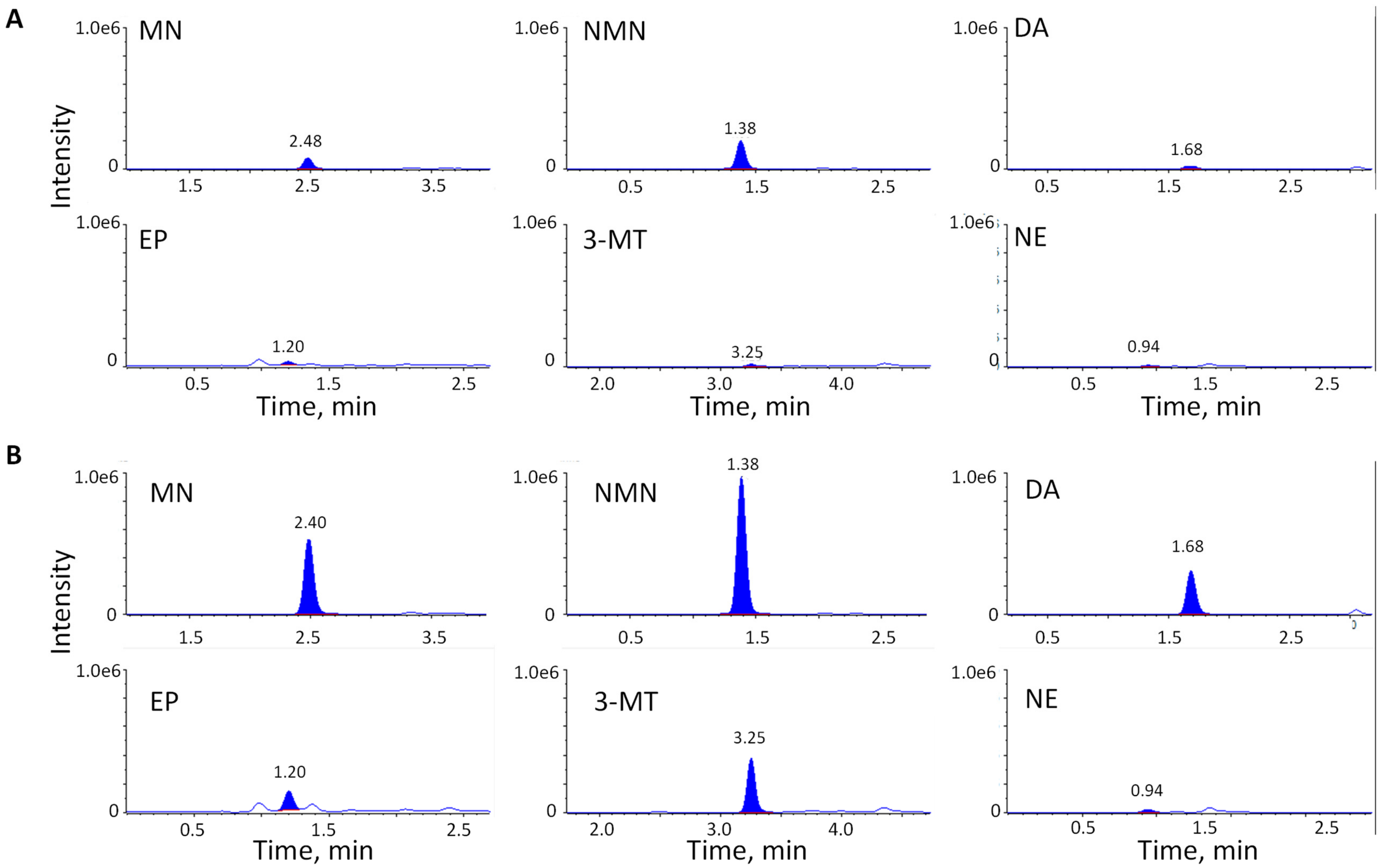

Supplementary Figure S2. Evaluation of peak resolution. Extracted ion chromatograms for metanephrine (MN), normetanephrine (NMN), dopamine (DA), epinephrine (EP), 3-methoxytyramine (3-MT), and norepinephrine (NE) in BioRad quality control level I (**A**, normal) and level II (**B**, abnormal) analyzed with dilute-and-shoot-liquid chromatography-mass spectrometry. The peak of interest is highlighted in blue, and the response is expressed as intensity (y-axis) vs. time in min (x-axis).

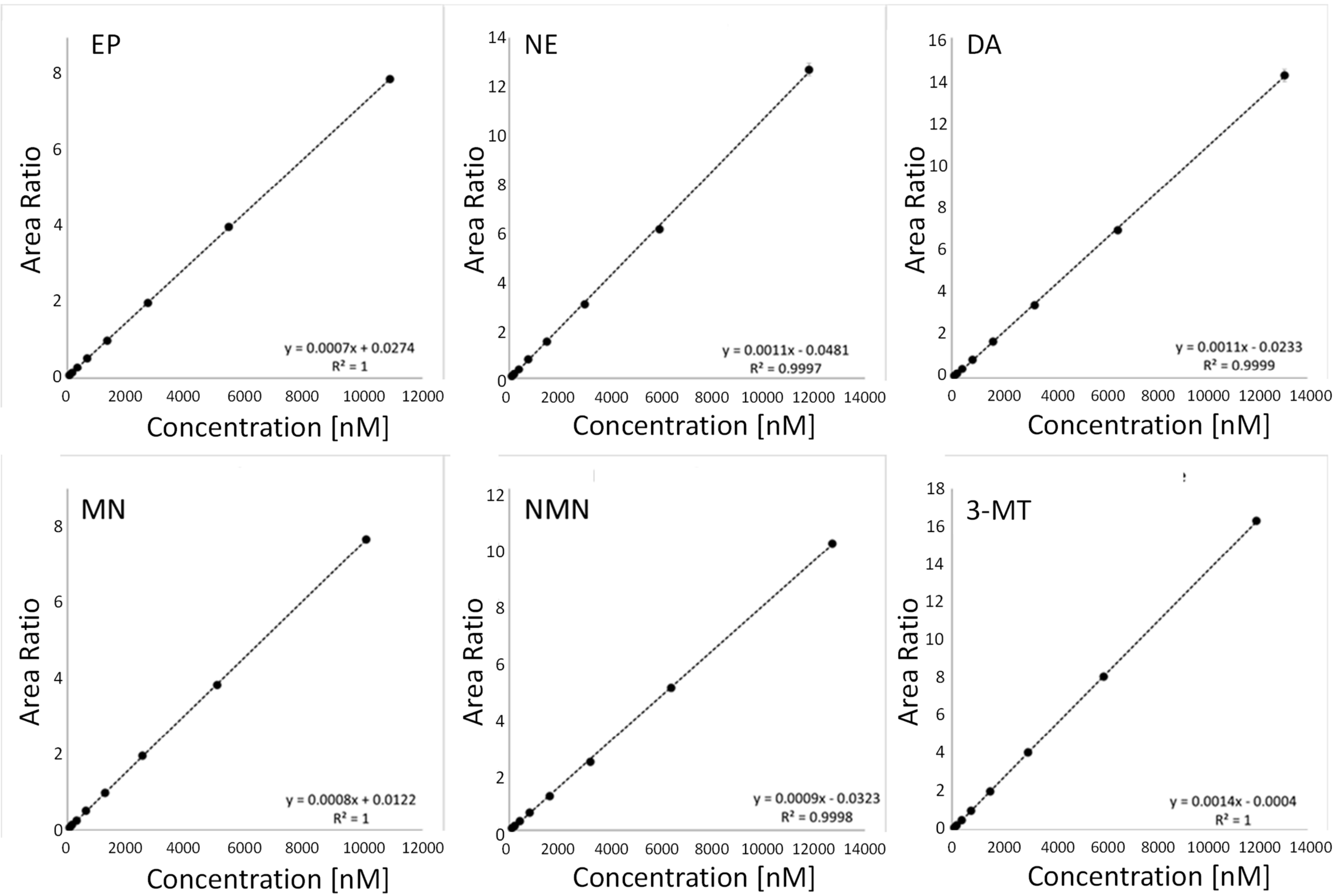

Supplementary Figure S3. Calibration standard curve. Urine calibration curves for the LC/MRM-MS analysis of metanephrine (MN), normetanephrine (NMN), 3-methoxytyramine (3-MT), epinephrine (EP), norepinephrine (NE), and dopamine (DA) over the concentration range of 79.2–10100, 85.3–10917, 93.5–11962, 85.3–10917, 92.3–11820, and 101.9–13055 nM, respectively. Standard curve linearity was assessed using the ratio of the analyte peak area to the IS area (y-axis) vs. concentration (x-axis) by weighted (1/x) linear regression analysis. The coefficient of determination (r^2) was found to be >0.9997. Values represent the means ± standard error of the mean (SEM) for n = 3 independent experiments.

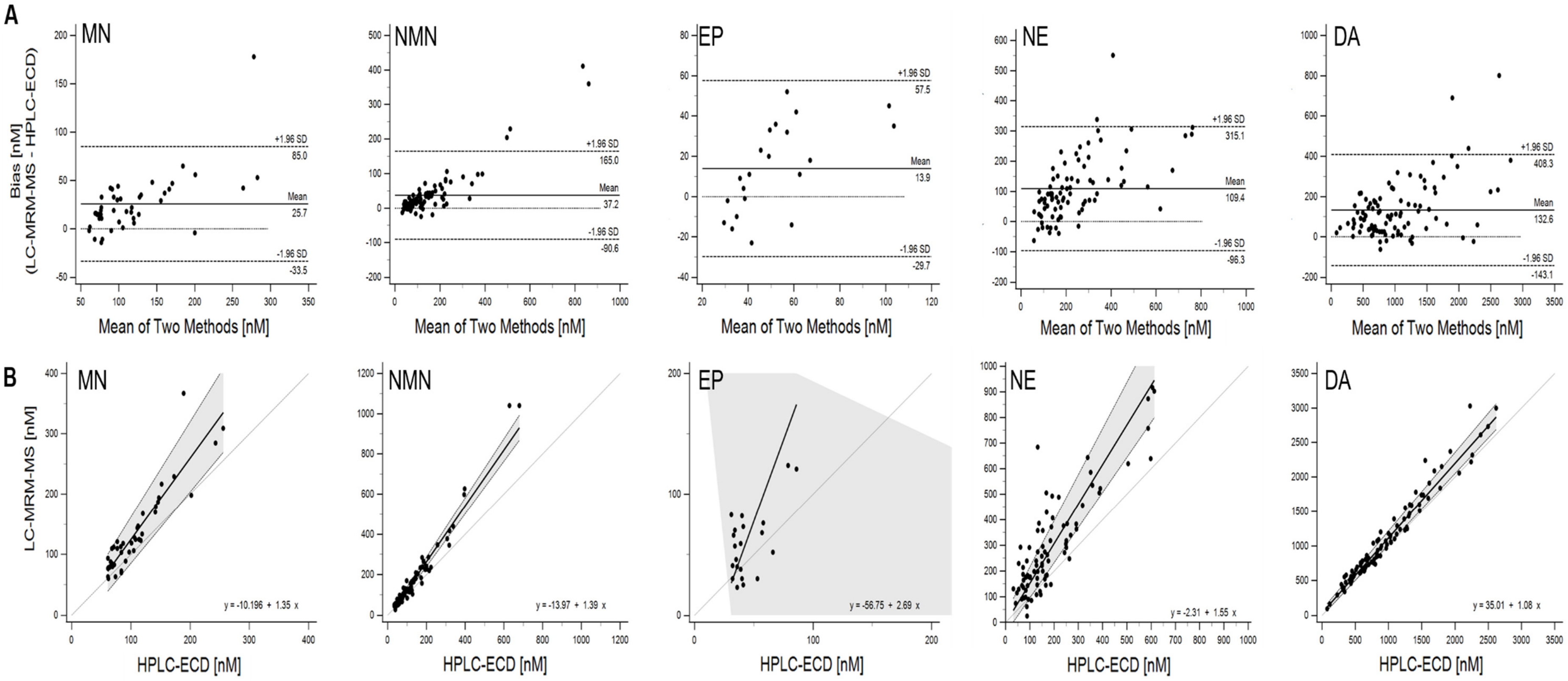

Supplementary Figure S4. Bland-Altman plot (A) and Passing-Bablok regression (B) for the results from the new LC/MRM-MS method versus the HPLC-ECD (y-axis), compared to the average results from the two methods (x-axis) for all analytes except for 3-MT which was not measured by the analytical reference method (Suppl. Table S2). Some samples were excluded from the analysis because they had a concentration below the LOQ of the analytical reference method (as majority of EP samples). (**A**) Horizontal lines are drawn at the mean difference (continuous line) and at the limits of agreement (dashed lines), which are defined as the mean difference plus and minus 1.96 times the standard deviation (SD) of the differences. The zero line is the line of equality, where the difference equals 0. (**B**) The identity line is grey. The black line shows the fitted regression line. The regression line equation is presented in the plots. The confidence intervals (95% CI) curve is the highlighted grey area.

Supplementary Tables

Supplementary Table S1. Accuracy assessment using EQA samples. QC samples obtained from the EQA (n=4) showed that the new LC-MS method performs very well, with a standard deviation index (SDI) of less than 2. 3-MT was not included in the QC samples.

Metanephrine				
	QC1	QC2	QC3	QC4
Lab results	996	__	1650	577
Group mean (n=12)	854	__	1787	528
SDPA	137	__	173.6	97
SDI	1.0	__	-0.8	0.5

Normetanephrine				
	QC1	QC2	QC3	QC4
Lab results	1980	2889	3500	1470
Group mean (n=11)	1862	2891	3164	1418
SDPA	360	441	482.8	216
SDI	0.3	0.0	0.7	0.2

Dopamine				
	QC1	QC2	QC3	QC4
Lab results	904	1220	1340	968
Group mean (n=11)	949	1244	1362	746
SDPA	218	224	245.2	134
SDI	-0.2	-0.1	-0.1	1.7

Epinephrine				
	QC1	QC2	QC3	QC4
Lab results	150	246	273	123
Group mean (n=13)	168	203	246	132
SDPA	38	40	48.8	26
SDI	-0.5	1.1	0.6	-0.3

Norepinephrine				
	QC1	QC2	QC3	QC4
Lab results	672	882	1000	418
Group mean (n=13)	742	906	1078	508
SDPA	153	166	197.9	93
SDI	-0.5	-0.1	-0.4	-1.0

*Abbreviations: QC: quality control; SDPA: Standard deviation of proficiency assessment; SDI: Standard deviation index.

Supplementary Table S2. Concentration values (nM) measured by HPLC-ECD and by LC-MRM-MS methods.

MN			NMN			EP			NE			DA		
Sample	ECD	MS	Sample	ECD	MS	Sample	ECD	MS	Sample	ECD	MS	Sample	ECD	MS
1	112	147	1	307	378	1	36	23	1	505	620	1	1521	1740
2	91	89	2	74	66	2	41	25	2	166	145	2	1469	1510
3	141	170	3	73	70	3	66	52	3	91	183	3	579	759
4	62	78	4	319	417	4	41	73	4	176	240	4	881	1200
5	105	106	5	258	348	5	40	30	5	358	535	5	1410	1780
6	77	110	6	173	184	6	58	76	6	248	306	6	2064	2060
7	111	144	7	59	67	7	53	30	7	94	90	7	597	769
8	152	217	8	39	26	8	32	30	8	172	264	8	291	446
9	84	70	9	112	115	9	57	68	9	131	172	9	975	1020
10	61	94	10	214	220	10	86	121	10	153	358	10	2243	2220
11	69	80	11	101	123	11	34	57	11	153	296	11	1345	1590
12	119	134	12	61	69	12	34	70	12	126	239	12	528	694
13	117	123	13	68	72	13	32	41	13	150	221	13	876	857
14	64	79	14	128	104	14	31	83	14	118	198	14	780	806
15	114	125	15	79	103	15	40	82	15	68	143	15	681	801
16	202	198	16	51	58	16	39	38	16	133	684	16	835	937
17	147	194	17	114	120	17	35	46	17	338	644	17	581	722
18	142	179	18	224	236	18	36	40	18	91	143	18	2386	2610
19	62	60	19	34	45	19	79	124	19	185	319	19	1025	1130
20	61	63	20	100	84	20	39	59	20	54	230	20	449	635
21	189	367	21	318	346	21	33	66	21	62	293	21	329	396
22	72	83	22	55	52				22	254	384	22	1931	2370
23	77	121	23	64	73				23	187	148	23	699	827
24	69	84	24	121	139				24	268	339	24	826	1070
25	97	104	25	94	129				25	251	291	25	1033	1060
26	84	73	26	125	122				26	243	272	26	1090	1100
27	87	118	27	194	225				27	386	506	27	1272	1270
28	66	81	28	87	122				28	101	178	28	843	855
29	256	309	29	59	64				29	251	318	29	777	753
30	84	103	30	102	128				30	75	96	30	445	500

31	66	88
32	146	187
33	71	112
34	67	86
35	173	229
36	68	110
37	106	128
38	61	77
39	83	113
40	243	285
41	120	168
42	101	119
43	108	125
44	74	63

31	52	51
32	118	130
33	202	228
34	52	57
35	98	170
36	83	98
37	133	176
38	60	57
39	200	241
40	117	158
41	48	78
42	155	205
43	41	53
44	48	55
45	143	184
46	75	97
47	62	65
48	73	87
49	62	99
50	72	95
51	629	1040
52	87	107
53	680	1040
54	76	82
55	39	57
56	102	119
57	134	165
58	78	94
59	339	438
60	51	63
61	34	52
62	81	135
63	178	284
64	186	265
65	60	77

31	168	506
32	153	200
33	131	147
34	239	376
35	54	129
36	94	179
37	84	176
38	138	386
39	82	99
40	150	228
41	67	92
42	588	873
43	88	239
44	598	640
45	101	174
46	72	214
47	133	358
48	72	187
49	169	430
50	98	292
51	588	758
52	73	136
53	32	131
54	74	165
55	127	222
56	263	248
57	47	115
58	42	75
59	133	274
60	291	383
61	166	182
62	189	372
63	145	109
64	89	26
65	219	489

31	964	969
32	569	605
33	957	1070
34	2496	2730
35	700	731
36	337	552
37	353	475
38	1282	1250
39	578	617
40	1303	1430
41	824	891
42	608	781
43	1037	1050
44	322	367
45	1031	1150
46	640	650
47	650	681
48	744	769
49	309	393
50	661	712
51	772	1040
52	2228	3030
53	801	739
54	75	95
55	2261	2320
56	880	969
57	339	341
58	1776	1840
59	478	565
60	1248	1230
61	815	840
62	1801	2150
63	633	639
64	361	579
65	672	862

	66	144	188		66	170	185	66	2620	3000
	67	43	54		67	88	62	67	1082	1390
	68	168	235		68	194	407	68	511	656
	69	88	110		69	149	258	69	616	662
	70	54	65		70	613	903	70	1121	1170
	71	176	157		71	292	363	71	115	161
	72	120	154		72	192	493	72	1249	1550
	73	41	37		73	317	456	73	1495	1740
	74	90	131		74	127	108	74	484	590
	75	81	108		75	83	126	75	1317	1470
	76	60	81		76	162	169	76	488	592
	77	89	129		77	605	917	77	436	557
	78	73	87		78	162	275	78	891	999
	79	397	626		79	133	198	79	1129	1290
	80	210	286		80	351	586	80	229	295
	81	145	209		81	390	523	81	1464	1600
	82	184	267		82	106	86	82	652	806
	83	88	101		83	142	121	83	1689	2090
	84	39	37					84	1598	1690
	85	129	169					85	605	661
	86	395	599					86	1614	1910
	87	124	167					87	435	458
	88	80	97					88	416	508
	89	181	238					89	851	1090
	90	145	180					90	1550	2240
	91	42	47					91	1320	1600
	92	63	64					92	957	1070
								93	1234	1390
								94	1174	1240
								95	1000	1180
								96	467	551

References

1. Lagerstedt SA, O'Kane DJ, Singh RJ. Measurement of plasma free metanephrine and normetanephrine by liquid chromatography-tandem mass spectrometry for diagnosis of pheochromocytoma. *Clin. Chem.*, 50(3), 603-611 (2004).
